# One‐Dimensional Acoustic Plasmons in Metallic Single‐Walled Carbon Nanotubes

**DOI:** 10.1002/nap2.70061

**Published:** 2026-04-08

**Authors:** GyeongPyo Jeon, Sihan Zhao, In‐Ho Lee, SeokJae Yoo

**Affiliations:** ^1^ Department of Physics Inha University Incheon Republic of Korea; ^2^ School of Physics Zhejiang Key Laboratory of Micro‐Nano Quantum Chips and Quantum Control and State Key Laboratory of Silicon and Advanced Semiconductor Materials Zhejiang University Hangzhou China; ^3^ Center for Quantum Technology Korea Institute of Science and Technology KIST Seoul Republic of Korea; ^4^ Department of Physics Program in Semiconductor and Device Research and Education on Next‐Generation Semiconductor Materials and Devices for Chiplet Technology and Physics Research Institute Inha University Incheon Republic of Korea

**Keywords:** carbon nanotubes, luttinger liquid, near‐field microscopy, plasmons

## Abstract

Acoustic plasmons, collective out‐of‐phase oscillations of image charges, can confine electric fields with exceptional strength, making them highly effective for applications requiring intense field localization. Here, we predict the existence of one‐dimensional (1D) acoustic plasmons in metallic single‐walled carbon nanotubes (SWNTs) on metallic substrates. These 1D acoustic plasmons achieve intensive electric field concentration with the plasmon wavelength contracted by approximately 22% compared to 1D plasmons in SWNTs. Because 1D plasmons in SWNTs are described by Luttinger liquid theory which captures the interactions among electrons in one‐dimensional systems, we anticipate that 1D acoustic plasmons can control quantum properties of the Luttinger liquid through optical and electrical means.

## Graphical abstract

Acoustic plasmons are collective out‐of‐phase charge oscillations that exhibit a sound‐like linear dispersion relation. They have been realized in metal‐insulator‐metal (MIM) structures, such as two‐dimensional graphene on a mirror. Here, we propose one‐dimensional (1D) acoustic plasmons and excitation schemes using a nanotube‐on‐a‐mirror geometry. The graphical abstract illustrates a detection scheme using infrared (IR) scanning near‐field optical microscopy (SNOM).

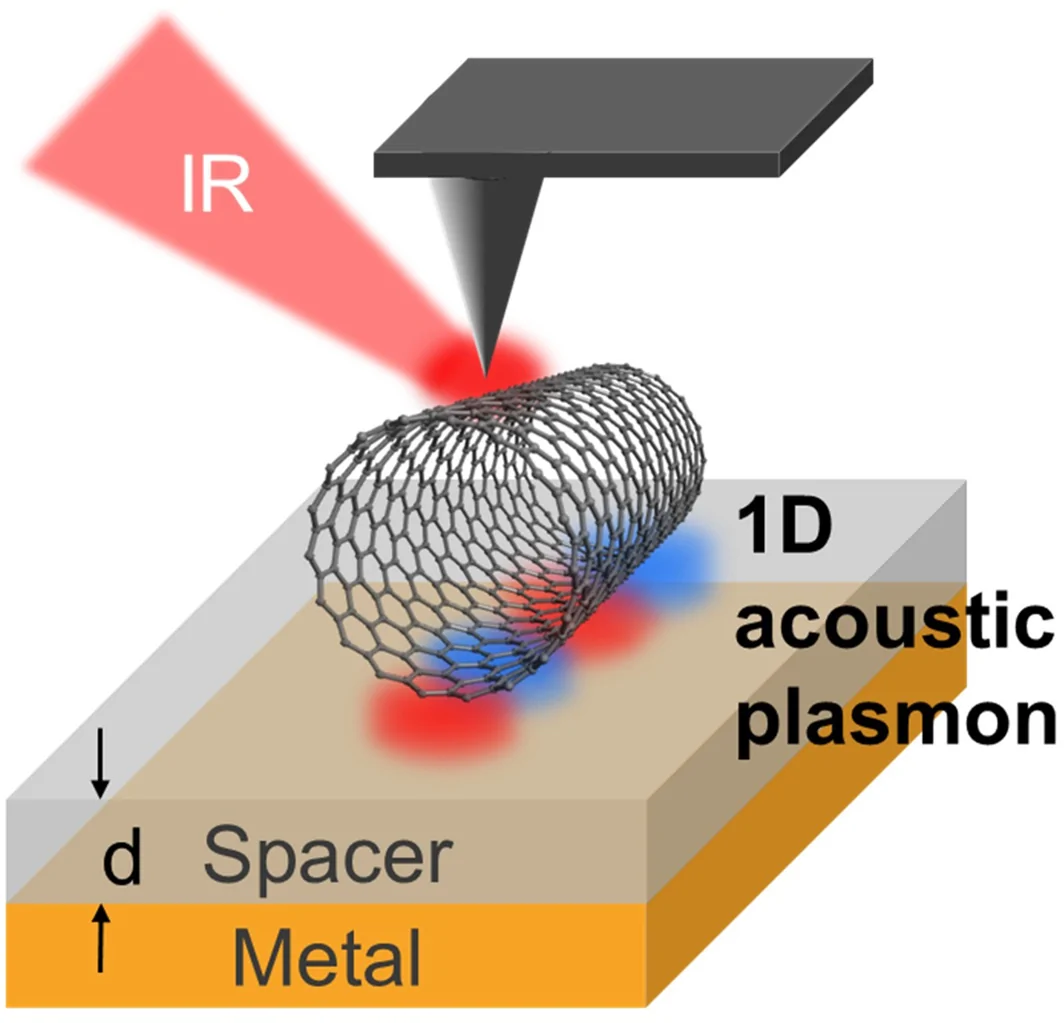

## 1 Introduction

Polaritons, quasiparticles arising from light–matter hybridization in van der Waals materials, have attracted significant interest due to their extreme spatial confinement and long temporal lifetimes [1]. Surface plasmon polaritons (SPPs), hybrid light‐electron quasiparticles in two‐dimensional (2D) graphene, exhibit SPP wavelengths up to ∼220 times shorter than the photon wavelength, with quality factors (*Q*) reaching up to ∼40 [2, 3, 4]. One‐dimensional (1D) SPPs in metallic [5] and semiconducting [6] single‐walled carbon nanotubes (SWNTs) experimentally provide even better SPP confinement (up to 100–1000) [6]; *Q* factors are up to 26 according to ref. [6], although theory suggests much larger *Q* in metallic SWNTs due to the forbidden backscattering of Dirac electrons [7].

The spatial confinement and temporal lifetime of SPPs can be further enhanced through coupling to image polaritons, enabled by a metallic substrate [8], a periodic metal array [9, 10], and spatially isolated structures [11, 12]. When 2D graphene is placed on a metallic substrate with an insulating spacer, image charges form in the metallic substrate. These image charges oscillate out‐of‐phase with the graphene plasmons, resulting in the emergence of a new plasmon mode, known as the acoustic graphene plasmon (AGP) mode. As its name suggests, the AGP barely couples to photons due to its out‐of‐phase oscillation nature. Its dispersion is also linear (*ω*∝*k* with the energy *ω* and the momentum *k*) as in acoustic waves, whereas bare graphene SPPs have quadratic dispersion (*ω*∝*k* ^2^) [13, 14, 15, 16]. Recent studies have reported that the AGP mode shrinks the plasmon wavelength by a factor of 2.3 compared to that of the GP mode. In addition, the AGP mode possesses better *Q* factor than the GP mode, which can be attributed to the faster enhancement of the huge plasmon momentum compared to the propagation length as the momentum increases [17]. AGPs also exhibit ultrastrong electric fields in the gap between graphene and the metallic substrate. Recent studies have exploited this capability for detecting the quantum surface response of metals [16], the quantum nonlocal effect [13], and sensing angstrom‐thick materials [8].

As acoustic modes in 2D graphene provide better spatial confinement and quality factors than bare graphene SPPs, the formation of acoustic modes in 1D SWNTs could potentially achieve previously unattainable fusion of ultrasmall modal volumes with high *Q* factors since these properties in 1D SWNT SPPs are, to a larger extent, superior to those in 2D graphene. The formation of acoustic plasmons in such 1D systems is expected to further enhance field confinement, providing a promising route toward subwavelength light control and sensing at the nanoscale. Interestingly, SWNTs follow the rules of Luttinger liquid theory, featuring intriguing spin–charge separated collective plasmon and spinon modes [18]. This further suggests that 1D acoustic plasmons in SWNTs may inherit these intriguing Luttinger liquid properties, while providing intense field localization and extremely low loss [5]. This exciting opportunity that 1D acoustic plasmons may endow has not yet been explored before, and it remains uncharted whether such fascinating 1D acoustic plasmon modes can be supported or not.

In this paper, we theoretically demonstrate that 1D acoustic plasmons can exist in SWNT on a metallic substrate with a thin insulating spacer, namely the SWNT‐on‐a‐mirror (NToM) geometry. We obtain an analytical expression for the 1D acoustic plasmon dispersion by solving an electromagnetic boundary value problem of the NToM geometry including the reflected near‐fields from the underlying metallic substrate. We further investigate the excitation of 1D acoustic plasmon mode with extreme field localization and propose two experimentally feasible excitation schemes: (i) scattering‐type scanning near‐field optical microscopy (s‐SNOM) and (ii) a grating coupler. We demonstrate that nanometer‐scaled tip of s‐SNOM can effectively excite the 1D acoustic plasmon mode of NToM in scheme (i) and periodic grating coupling can also efficiently excite these modes in scheme (ii), both of which confirm the feasibility of far‐field excitation and detection of 1D acoustic plasmons. We anticipate that the unique combination of properties in SWNTs and acoustic plasmons opens new opportunities toward deep‐subwavelength optics as well as quantum optoelectronics based on engineerable Luttinger liquid plasmons.

## 2 Result

### 2.1 1D Acoustic Plasmon Dispersion

To obtain the dispersion of the 1D acoustic plasmon, we model the SWNT as a solid cylindrical waveguide with radius R and the relative permittivity *ε*_SWNT_ (Supporting Information S1: Figure S1). We use the relative permittivity of a bare SWNT *ε*_SWNT_ satisfying the following equation:

(1) $$\text{Re}\left\{\varepsilon_{\text{SWNT}}\left(\omega \right)\right\}=-\frac{16}{1-g^{2}}\frac{k_{e}e^{2}}{\varepsilon_{\text{med}}\hbar v_{F}}\frac{v_{F}^{2}}{\pi R^{2}}\frac{1}{\omega^{2}}$$

which follows the Luttinger liquid physics [19]. *g* is the Luttinger liquid interaction parameter of a bare SWNT. $k_{e}=1/4\pi \varepsilon_{0}$ and ℏ is the reduced Planck constant. $\text{Im}\left[\varepsilon_{\text{SWNT}}\left(\omega \right)\right]$ can be obtained by the relation $Q=\left\{\omega \text{Re}\left(d\varepsilon_{\text{SWNT}}/d\omega \right)\right\}/${$\text{Im}\left(\varepsilon_{\text{SWNT}}\right)$} for a given *Q* = 20 [20]. *v*_F_ = 8 × 10^5^ m/s is the Fermi velocity. The cylinder is embedded in a medium with the relative permittivity *ε*_med_. To induce image charges creating the acoustic plasmon mode, NToM geometry is used in this work; the SWNT is placed on an insulator of thickness d, which is placed on the metal substrate. Because plasmon modes of SWNTs can be found at infrared frequencies [5], we model the metallic substrate as a perfect electric conductor (PEC). In this configuration, charge oscillations in the SWNT couple strongly to their image charges with opposite signs in the metal, resulting in the acoustic plasmon mode. The resulting out‐of‐phase charge oscillation of acoustic plasmons increases the plasmon momentum and consequently slows down the plasmon velocity, ${\;v}_{p}/c=k_{0}/k_{p\;}$ ≪ 1 where c is the speed of light and $k_{0}$ is the free‐space wavevector. Figure 1a shows the schematic drawing of 1D acoustic plasmons realizable by the NToM geometry, and the electric field is strongly confined in the gap (with size *d*).

**FIGURE 1:**
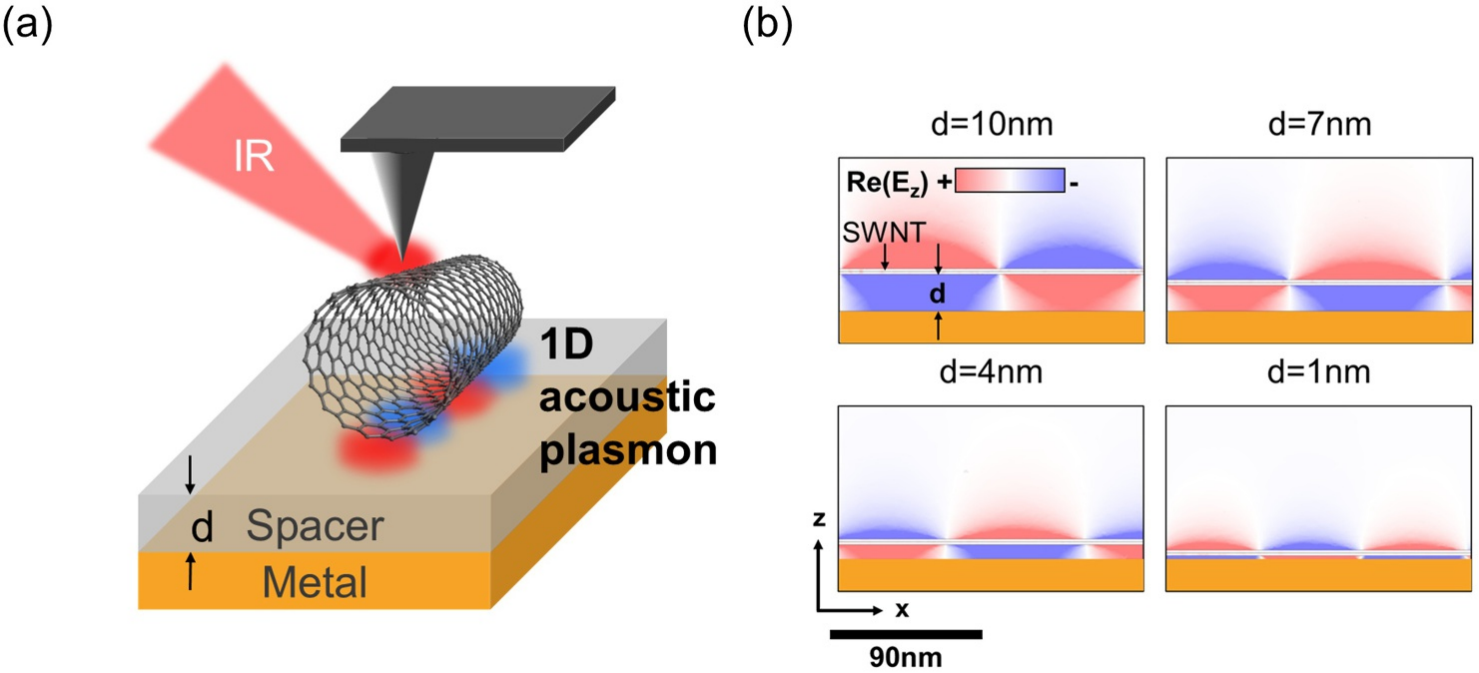
(a) Schematic drawing of a 1D acoustic plasmon supported by a single‐walled carbon nanotube (SWNT) placed above a metallic substrate with an insulator of thickness *d*. (b) Numerically simulated spatial profiles of the electric field, Re(E_z_) (*x*, *z*), at *λ* = 10.6 μm, demonstrating the dependence of plasmon confinement on different values of *d* for an SWNT with radius *R* = 0.75 nm.

In Figure 1b, we find that the gap size can change the acoustic plasmon wavelength significantly; the acoustic plasmons are slowed down as the gap size is reduced. To predict the acoustic plasmon mode analytically, we solve the electromagnetic boundary value problem on the SWNT surface, considering the reflection of the plasmonic near‐fields from the metallic substrate. Using the angular spectrum method, which we used for a bare SWNT in our previous work [21], we obtain the plasmon dispersion relation of the NToM,

(2) $$\frac{\varepsilon_{\text{SWNT}}}{\varepsilon_{\text{med}}}\frac{k_{\rho ,\text{med}}}{k_{\rho ,\text{SWNT}}}=-\frac{I_{0}\left(k_{\rho ,\text{SWNT}}R\right)}{I_{1}\left(k_{\rho ,\text{SWNT}}R\right)}\frac{K_{1}\left(k_{\rho ,\text{med}}R\right)-RW_{0}\left(k_{\rho ,\text{med}}R,k_{\rho ,\text{med}}d\right)}{K_{0}\left(k_{\rho ,\text{med}}R\right)+RW_{0}\left(k_{\rho ,\text{med}}R,k_{\rho ,\text{med}}d\right)}$$

Here, the radial momentum inside (outside) the SWNT is denoted by $k_{\rho ,\text{SWNT}\left(\text{med}\right)}=\sqrt{k_{p}^{2}-k_{\text{SWNT}\left(\text{med}\right)}^{2}}$, where $k_{\text{SWNT}\left(\text{med}\right)}=\sqrt{\varepsilon_{\text{SWNT}\left(\text{med}\right)}}k_{0}$ is the wavenumber in the media of the relative permittivity $\varepsilon_{\text{SWNT}\left(\text{med}\right)}$. ${\;k}_{p}$ is the wavenumber of plasmon which propagate in the axial direction of the SWNT. *k*_0_ = 2π/*λ*_0_ is the wavenumber in air, where *λ*_0_ is the wavelength of light in free space. $I_{n}$ represents the *nth* order modified Bessel function of the first kind, and $K_{n}$ represents the *nth* order modified Bessel function of the second kind. $RW_{0}\left(k_{\rho ,\text{med}}R,k_{\rho ,\text{med}}d\right)$ is the 0‐th order modified Bessel function representing the potential of the reflected near‐field from the metallic substrate, that is, the potential perturbation by the image charge formed in the substrate. When *RW*_0_ vanishes, Equation (2) reduces to the plasmon dispersion of SWNTs [19]. The plasmon wavelength $\lambda_{p}$ and the quality factor *Q* are obtained by solving Equation (2) for the complex‐valued $k_{p}=2\pi /\lambda_{p}\left(1+i/Q\right)$.

Figure 2a demonstrates numerically and analytically calculated dispersions of NToM and bare SWNT. The bare SWNT refers to a SWNT embedded in a dielectric medium without any metallic substrate. To calculate the dispersion of bare 1D plasmons freestanding in air, we use experimentally obtained parameters of metallic SWNTs in Equation (1); the Luttinger liquid parameter is chosen to be *g* = 0.31, providing the plasmon wavelength of 90 nm for a SWNT of radius *R* = 0.75 nm at the photon wavelength of 10.6 μm. *g* = 0.31 for metallic SWNT has also been confirmed by electrical transport experiments [22]. The relative permittivity of the background medium is $\varepsilon_{\text{med}}=1$. For 1D acoustic plasmons of NToM, we use Equation (2) to obtain the dispersion, while keeping other parameters the same, for example, *ε*_SWNT_, *R*, and *g*. $RW_{0}\left(k_{\rho ,\text{med}}R,k_{\rho ,\text{med}}d\right)$ was evaluated using the resonant momentum approximation used in our previous work [21].

(3) $$RW_{0}\left(k_{\rho ,\text{med}}R,k_{\rho ,\text{med}}d\right)\approx \frac{1}{4}r\left(k_{\rho ,\text{med}}R\right)\left(e^{-k_{\rho ,\text{med}}\left(2d-R\right)}+e^{-k_{\rho ,\text{med}}\left(2d+R\right)}\right)$$

representing reflection of plasmonic near‐fields by the metallic substrate. In. Figure 2, numerical simulations were performed using the electromagnetic waves frequency domain (EWFD) module in COMSOL multiphysics. To reduce the computational load, an air gap was introduced instead of placing an insulator layer on top of the metal (Au) substrate. All numerical simulations employed metal (Au) substrate rather than a PEC boundary condition. A point dipole source excites SWNT plasmons at the end of the SWNT. It is located at 140 nm above the SWNT surface. Then, we extract the plasmon wavelengths (Figure 2a) and *Q* factors (Figure 2b) from the electric field component normal to the SWNT surface (*d* = 2 nm used in Figure 2). As the SWNT approaches the metal surface (i.e., a decrease in the insulator thickness d), the effective permittivity $\varepsilon_{\text{med}}$ experienced by the SWNT cannot be determined analytically due to the nonuniform dielectric environment. Therefore, $\varepsilon_{\text{med}}=1.2$ was obtained by fitting the analytical results to the numerical simulations.

**FIGURE 2:**
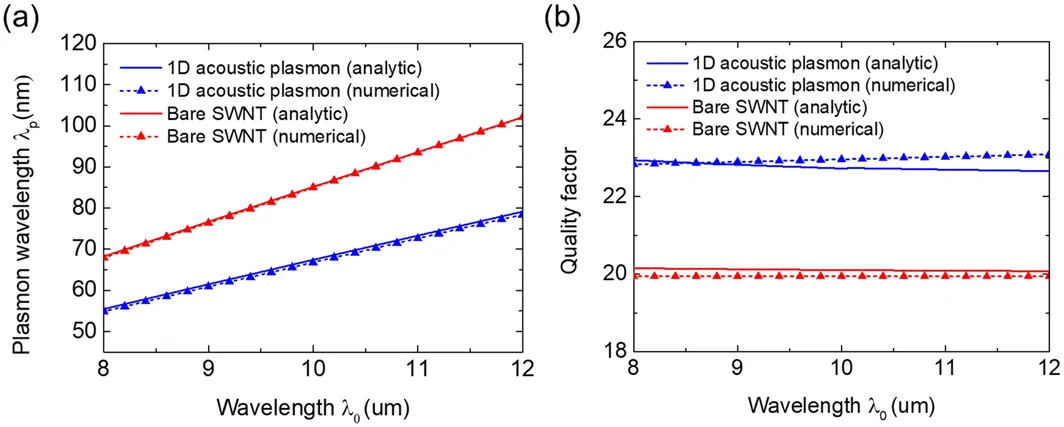
(a) Plasmon wavelength reduction in the proximity of metal (blue lines) compared to a bare SWNT (red lines) in terms of the wavelength of light $\lambda_{0}$. (b) Enhancement of the quality factor Q in the acoustic plasmon mode. The blue lines represent the 1D acoustic plasmons while the red lines correspond to bare SWNT plasmons. Solid lines denote analytical calculations, and dashed lines with triangular markers indicate numerical simulations. In both (a) and (b), the analytic and numerical results were calculated for an insulator of thickness *d* = 2 nm.

As shown in Figure 2, our analytic model agrees well with the numerical simulation, successfully capturing both the plasmon wavelength contraction and the dispersion. In Figure 2a, the dispersion of 1D acoustic plasmons exhibits a linear shape, the same as that of the Luttinger liquid plasmon dispersion in bare SWNTs [23]. 1D acoustic plasmons of NToM show the significant contraction of the plasmon wavelength by ∼22% compared to 1D plasmons of the bare SWNT for *d* = 2 nm. In Figure 2b, we find that the quality factor *Q* is enhanced in the acoustic plasmon mode. It has been reported that bare SWNTs already have a high *Q* factor (∼20 according to infrared experiments [5]) because the Dirac electrons residing in metallic SWNTs fundamentally forbids backscatterings due to the smoothly varying charged impurity potential [24, 25] and thermally activated phonons [26]. 1D acoustic plasmons of NToM with high plasmon momentum $k_{p}$ further suppress radiative loss that couples to the free space propagation with the photon momentum $k_{0}$ ≪$k_{p}$. This results in the *Q* enhancement of the acoustic plasmon mode shown in Figure 2b.

### 2.2 Comparison Between 1D Acoustic Plasmon and GPs

To compare the characteristics of 1D acoustic plasmons, bare SWNT plasmons, and GPs, we calculate their dispersions in Figure 3a. The dispersion of graphene was calculated by solving the electromagnetic boundary condition at the 2D conducting sheet [27]. The dispersion is expressed as follows:

(4) $${n_{2}}^{2}k_{1z}+{n_{1}}^{2}k_{2z}+i\eta_{0}\sigma \frac{k_{1z}k_{2z}}{k_{0}}=0$$

where $k_{0}=\frac{2\pi }{\lambda }$, $k_{1z}=\sqrt{{k_{p}}^{2}-{\left(n_{1}k_{0}\right)}^{2}}$ and $k_{2z}=\sqrt{{k_{p}}^{2}-{\left(n_{2}k_{0}\right)}^{2}}$. Here $n_{1}$ and $n_{2}$ denote the refractive indices above and below the conducting layer, respectively. σ is the optical conductivity of graphene, and $\eta_{0}$ is the free‐space impedance. The optical conductivity of graphene is determined by the sum of interband and intraband contributions, $\sigma =\sigma_{\text{inter}}+\sigma_{\text{intra}}$, whose expressions are given by the following equation [28, 29]:

(5) $$\sigma_{\text{intra}}\left(\omega \right)=\frac{2e^{2}k_{B}T}{\pi \hbar^{2}}\ln\left[2\;\cos\;h\left(\frac{\mu }{2k_{B}T}\right)\right]\frac{i}{\omega +i/\tau }$$

(6) $$\sigma_{\text{inter}}\left(\omega \right)=\frac{e^{2}}{4\hbar }[H\left(\frac{\omega }{2}\right)+i\frac{4\omega }{\pi }\int_{0}^{\infty }d\varepsilon \frac{H\left(\varepsilon \right)-H\left(\frac{\omega }{2}\right)}{\omega^{2}-4\varepsilon^{2}}$$

(7) $$H\left(\varepsilon \right)=\frac{\sin\;h\left(\hbar \varepsilon /k_{b}T\right)}{\cos\;h\left(\mu /k_{b}T\right)+\cos\;h\left(\hbar \varepsilon /k_{b}T\right)}$$

respectively. Temperature and relaxation time are chosen to be T=300K and τ=100fs, respectively [21]. For a fair comparison to a bare SWNT freestanding in air (solid blue and red lines in Figure 3), we choose the refractive indices *n*_1_ = *n*_2_ = 1. The dashed lines in Figure 3 represent GPs for different Fermi energies E_f_ = 0.12, 0.16, 0.2, and 0.24 eV. The GPs show quadratic dispersion (*ω*∝*k* ^2^), which is a typical feature of optical plasmons. In Figure 3, as $E_{f}$ increases, the plasmon wavenumber $k_{p}$ of GPs decreases while the quality factor Q increases, indicating reduced confinement but improved propagation. Fermi energy *E*_*f*_ which is associated with electrical gating and chemical doping [4, 30], plays a crucial role in determining the plasmonic field localization and the plasmon loss. Graphene undergoes severe Landau damping caused by the interband transition when E_f_ is less than half of the excitation energy [27]; a lower *E*_*f*_ yields stronger localization but a lower *Q*, representing a fundamental trade‐off between the field localization and loss. To achieve a high‐quality factor in GPs, a relatively high gating level is therefore needed. In contrast, 1D acoustic plasmons in SWNTs naturally appear without applying a gate voltage, owing to the carrier density independence in the Luttinger liquid system in SWNT [31], yet still provide strong field confinement and a quality factor surpassing (or at least comparable to) that of GPs. Their intrinsic ability to propagate along the SWNT with minimal attenuation and extreme electric‐field localization highlights the advantage of 1D acoustic plasmons as a practical and robust platform for plasmonic applications. Moreover, a recent study has reported a tunable Luttinger liquid system in 1D that sensitively depend on carrier doping [32], further suggesting the potential of tunable 1D acoustic plasmons.

**FIGURE 3:**
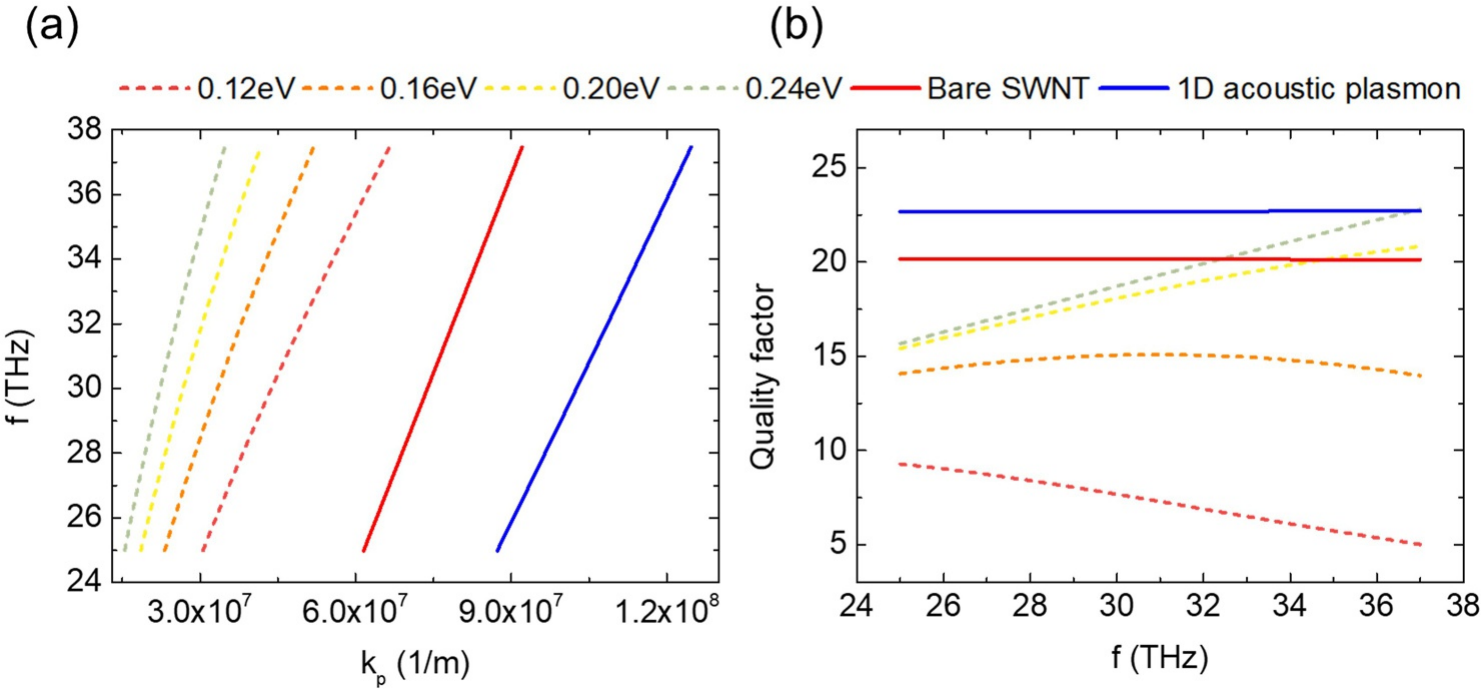
(a) Dispersion relation and, (b) quality factor Q of 1D acoustic plasmon (solid blue line), bare SWNT plasmon (solid red line), and GPs for Fermi energies *E_f_* = 0.12, 0.16, 0.20, and 0.24 eV (dashed lines). In Figure 3, the insulator thickness is *d* = 2 nm for the 1D acoustic plasmon.

### 2.3 s‐SNOM Excitation of 1D Acoustic Plasmon

The large momentum mismatch between 1D plasmons and photons makes their far‐field plane wave excitation challenging, and thus it demands more advanced excitation techniques [5]. Here, we propose two excitation schemes: scattering‐type scanning near‐field optical microscopy (s‐SNOM) and a grating coupler. In the first scheme using s‐SNOM, sharp metallic tips are employed to detect the near‐field intensity of the sample sensitively. We demonstrate that 1D acoustic plasmons can be excited by the s‐SNOM technique using numerical simulations, although the tip‐SWNT coupling should be carefully considered in the NToM geometry because the plasmon–photon momentum mismatch is even larger in the 1D acoustic plasmons than optical plasmons of a bare SWNT.

In s‐SNOM, the tip concentrates the incident light into localized evanescent fields that excite 1D plasmons in the SWNT, whereas the backscattered near‐field signal from the tip reveals their spatially resolved propagation forming a standing wave. To confirm the feasibility of 1D acoustic plasmon excitation and detection, we numerically calculate the electric field intensity at the tip end while the tip scans across the SWNT axis. Figure 4a illustrates the schematic of this process: the tip scans along the axial direction of the SWNT (*x*‐axis), launching 1D acoustic plasmons that propagate along the tube and reflect at the physical edge of the SWNT [33]. As a result, an electric field is localized between the tip and the edge region which facilitates the detection of 1D acoustic plasmons. The tip‐launched plasmons denote those excited at the tip apex by the localized high‐momentum near field, which propagate along the SWNT axis [5]. Upon reaching the end of the SWNT, these plasmons are reflected and travel back toward the tip as edge‐reflected plasmons. The interference between the tip‐launched and edge‐reflected plasmons produces a standing‐wave resonance pattern whenever the tip‐to‐edge distance corresponds to integer multiples of half the plasmon wavelength, *λ*_*p*_/2 [33]. As the tip scans along the SWNT, the near‐field signal recorded by s‐SNOM follows this oscillating standing‐wave field profile, directly visualizing the 1D acoustic plasmons in SWNTs.

**FIGURE 4:**
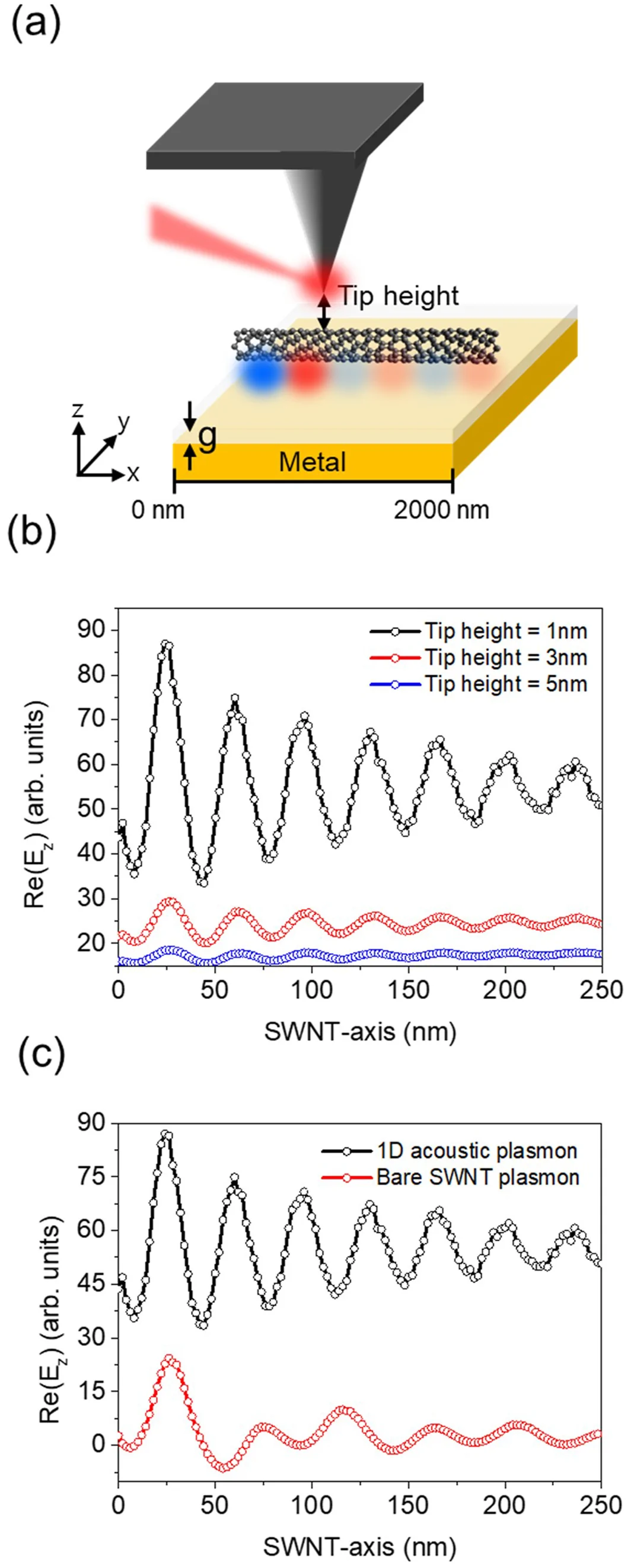
(a) Schematic drawing of 1D acoustic plasmon excitation by s‐SNOM (scattering type scanning near‐field microscopy), (b) near‐field measurement of 1D acoustic plasmon with respect to the tip heights of 1 nm (the black line), 3 nm (the red line), and 5 nm (the blue line). The tip scans from the left to the right end of the SWNT along its axial direction. (c) Comparison of the electric field Re(E_z_) of 1D acoustic plasmon (the black line) and bare SWNT plasmon (the red line) at the same tip height of 1 nm.

Figure 4b shows the simulated near‐field amplitude Re(E_z_) at the tip apex as a function of the position along the SWNT axis (*x* direction) for different tip‐SWNT distances (*z* direction). As the distances increase from 1 to 5 nm, the magnitude of Re(E_z_) decreases exponentially, indicating the evanescent decay of the acoustic plasmon field away from the SWNT surface. This behavior explains why the tip must be placed sufficiently close to the SWNT to both excite and detect the 1D acoustic plasmons due to the evanescent nature of the plasmon fields. Our simulated oscillatory field distribution in Figure 4b exhibits periodic peaks separated by half of the plasmon wavelength, consistent with previous near‐field studies of GPs [4, 19].

Figure 4c compares the simulated electric field distributions of the 1D acoustic plasmon and bare SWNT plasmon along the SWNT axis at a tip‐SWNT distance of 1 nm. The 1D acoustic plasmon exhibits significantly stronger field localization and larger oscillation amplitude than the bare SWNT plasmon. Because bare SWNT plasmons can be captured by s‐SNOM [5, 28], the enhanced near‐field signal of the 1D acoustic plasmon indicates that it can be even more readily detected in s‐SNOM measurements.

Another interesting feature of 1D acoustic plasmons (Figure 4c) is that even‐order peak suppression caused by plasmon diffraction is barely observable. When the plasmon traveling along a SWNT reaches the end, it is reflected backward, some evanescent waves are diffracted toward the air outside the SWNT [33, 34]. As a result, the reflected plasmons gain a finite phase shift (∼0.2π) with respect to the incoming plasmons. This phase shift upon the plasmon reflection causes constructive interference at odd peaks and destructive interference at even peaks, giving rise to the characteristic even–odd peak oscillation that can be found in s‐SNOM measurements for graphene plasmons and Luttinger liquid plasmons in SWNTs [33, 34] as shown in Figure 4c. However, in our NToM geometry the 1D acoustic plasmons are extremely confined so that the diffracted evanescent field decays rapidly and cannot extend far enough to interfere with the propagating 1D acoustic plasmons (Supporting Information S1: Figure S2). As a result, the even–odd oscillation disappears in acoustic plasmons. This behavior can be further confirmed by the effect of tip height. Unlike the 1D optical plasmons, where the even–odd peak oscillation becomes more pronounced as the tip moves away [34], the 1D acoustic plasmons in our NToM structure show no noticeable variation with different tip heights (Figure 4b). The absence of even–odd peak oscillation is a critical signature of acoustic plasmons, distinguishing them from 1D and 2D optical plasmons [34].

However, despite the clear feasibility in our numerical simulation, practical s‐SNOM detection of 1D acoustic plasmons poses a challenge. Because of the highly confined evanescent nature of 1D acoustic plasmons, the tip must be positioned within only a few nanometers from the SWNT surface to effectively launch and detect the plasmons. This extreme proximity requirement makes the measurement highly sensitive to tip conditions, drift, and sample roughness, often resulting in unstable signals and reduced reproducibility. To overcome these challenges, alternative coupling schemes such as grating excitation may provide an effective route. In a grating‐based geometry, incident infrared light can be directly coupled into the 1D acoustic plasmon through scattering from the grating edge, enabling 1D acoustic plasmon excitation without the tip.

### 2.4 Grating Excitation of 1D Acoustic Plasmon

Another widely used geometry for the plasmon excitation is the grating structure, which couples light into a plasmon mode by a momentum kick from the patterned periodic structures such as grating [8, 9, 35, 36, 37]. The 1D acoustic plasmon resonator consists of a continuous SWNT placed on a periodic metal ribbon array with a nanoscale gap *a* filled with alumina (Figure 5a). Each metal ribbon has a width *w*, spacing *s*, and periodicity *p = w + s*. An optical spacer with thickness *d* made of amorphous silicon (*n*_s_ = 3.3) and a back reflector are also incorporated beneath the structure to enhance the coupling between the incident TM‐polarized wave and the 1D acoustic plasmons.

**FIGURE 5:**
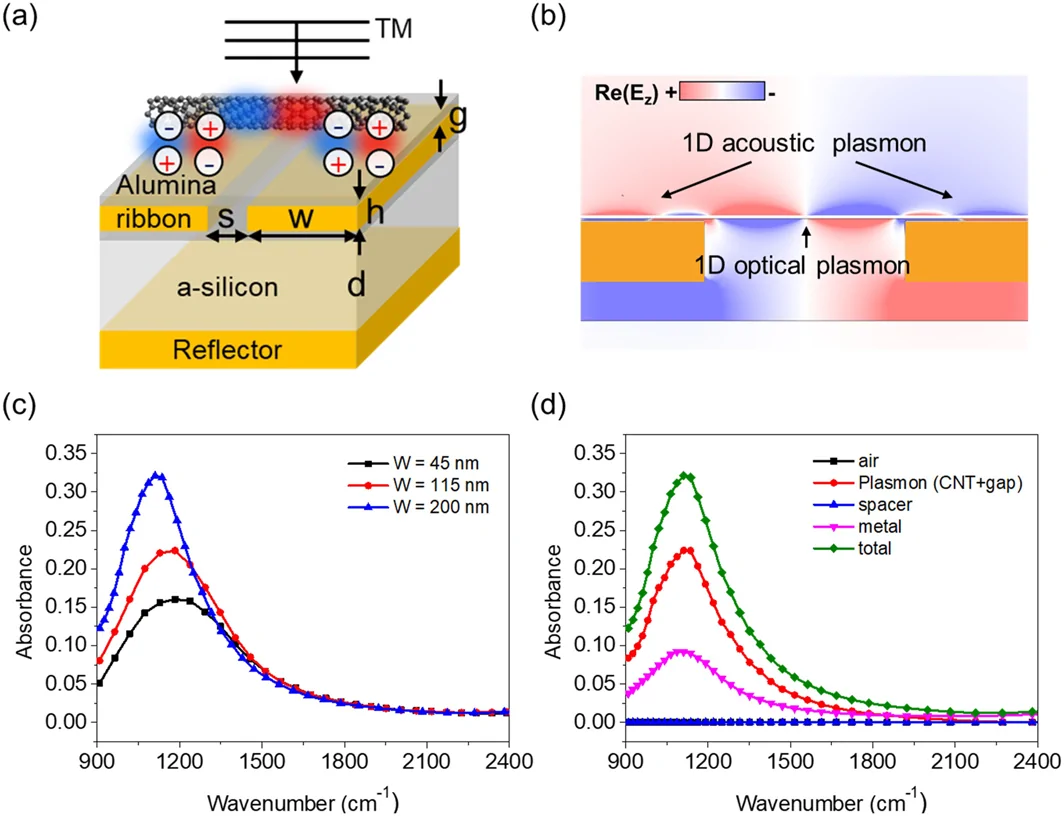
(a) Schematic drawing of 1D acoustic plasmon excitation by grating with reflector. (b) Electric field profile of the 1D acoustic plasmon and bare SWNT plasmon. (c) Numerical results of the 1D acoustic plasmon resonator with an optical spacer and reflector. (d) Absorption contributions from the distinct parts of the resonator with *w* = 200 nm. In all cases, we used alumina as a gap‐filling material with a gap size of *a* = 2 nm. The spacer is amorphous silicon (a‐silicon) with thickness *d* = 580 nm, the spacer index *n*_s_ = 3.3 and s is fixed to be 20 nm except for the case of (b) which s is 100 nm.

Figure 5b shows the numerically calculated field profile of the 1D acoustic plasmons coupled to the grating. The SWNT above the metal ribbons supports 1D acoustic plasmons (i.e., the shorter plasmon wavelength), whereas the metal‐free region supports conventional 1D optical plasmons (i.e., the longer plasmon wavelength). Although the plane wave cannot excite 1D plasmons, Figure 5b demonstrates that the plane wave can efficiently excite both acoustic and optical plasmons in the SWNT. This efficient far‐field excitation results from the momentum kick provided by the grating [8]; the periodic grating provides the additional momentum to incoming photons through scattering events from the edge of the metal ribbon, allowing coupling to high‐momentum plasmons.

In Figure 5c, we calculate the absorbance of the whole structure including the grating, SWNT, spacer, and back‐reflector. As we increase the ribbon width from *w* = 45 nm, to 115 nm and 200 nm, the resonance peak redshifts, whereas absorbance is enhanced for larger *w*. This strong absorption response enables the excitation and detection of 1D acoustic plasmons through far‐field experiments.

To further clarify the origin of this absorbance, we examine the absorbance contributions from each component of the structure in Figure 5d. Absorbance contributions are obtained by the ohmic loss calculation, $Im\left(\epsilon \right)\int {\left|E\right|}^{2}dV/2$, where ***E*** denotes the electric field inside each region, respectively. Figure 5d shows that most of the photon energy is used to excite 1D acoustic and optical plasmons in SWNT (the red curve), whereas some energy is dissipated as ohmic losses in the metallic ribbon grating and the back‐reflector (the magenta curve). Because the spacer is dielectric, its absorbance contribution is negligible (the blue curve).

Our results in Sections 2.3 and 2.4 suggest two experimental pathways for verifying 1D acoustic plasmons. Near‐field imaging with s‐SNOM (Section 2.3) enables direct visualization of plasmon interference patterns along the SWNT, whereas the grating coupler (Section 2.4) can harvest conventional far‐field spectroscopy to capture the spectral signatures of 1D acoustic plasmons.

## 3 Discussion

Before concluding, it is worthwhile to mention the possible transformation of the Luttinger liquid parameter *g* in the 1D acoustic plasmon mode. In Luttinger liquid theory, the slope of the dispersion relation is related to *g*, $\omega =\left(v_{F}/g\right)k_{p}$. The g parameter for bare SWNTs has been reported to be approximately 0.31 [22], whereas our result shows a slightly reduced slope which may suggest an effectively larger value of about 0.38 for 1D acoustic plasmons in *d* = 2 nm gap. This could imply weaker electron–electron interactions due to the screening by the nearby metal. Because the Luttinger liquid parameter *g* depends on the size of the gap and tends to increase as the gap is reduced, the observed change might be qualitatively consistent with such screening effects. The 1D acoustic plasmon can be understood as the screened Luttinger liquid plasmon [38] because one mode is localized in the SWNT, whereas the other is induced in the metallic substrate. In this sense, the metal screening effect is already incorporated into the dispersion of the 1D acoustic plasmon in Equation (1) through $RW_{0}$, giving rise to possible route to extend the same g relation with plasmon velocity to this hybridized mode [38].

## 4 Conclusion

To summarize, we have predicted the existence of a one‐dimensional (1D) acoustic plasmon mode in SWNTs placed on the metallic substrate with an insulating spacer. Our theory shows that the plasmon wavelength decreases and that the 1D plasmon is highly confined in the gap with suppressed radiative losses. We have found that the plasmon wavelength of 1D acoustic plasmons varies depending on the size of the gap. We have also proposed excitation schemes: (i) near‐field excitation and detection using s‐SNOM and (ii) far‐field coupling by grating. They provide experimental routes to detect these modes experimentally. This work opens opportunities to explore acoustic plasmons in 1D SWNTs, whereas conventional efforts have been devoted to 2D acoustic plasmons in graphene. The combination of the unique properties of 1D plasmons with the acoustic mode, together with the practical excitation strategies outlined here, will enable control and utilization of novel properties of 1D Luttinger liquid plasmons in SWNTs.

## Author Contributions

All authors have accepted responsibility for the entire content of this manuscript and approved its submission. All authors have given approval to the final version of the manuscript. G.J, S.Z. and S.Y. wrote the manuscript. S.Y., S.Z., and I.‐H.L. conceived the project. G.J. and S.Y. derived equations for 1D acoustic plasmon. G.J. performed numerical calculations.

## Funding

This work was supported by the National Research Foundation of Korea (NRF) grant funded by the Korea Government (MSIT) (No. RS‐2023‐00254920, No. RS‐2025‐16067780).

## Conflicts of Interest

The authors declare no conflicts of interest.

## Supporting information


Supporting Information S1

## Data Availability

All data generated or analyzed during this study are included in this published article and its supplementary information files.
