# Supplementary material for: One‐Dimensional Acoustic Plasmons in Metallic Single‐Walled Carbon Nanotubes
Source: Nanophotonics. 2026 Apr 8;15(7):e70061. doi: 10.1002/nap2.70061 (PMC13642575; doi:10.1002/nap2.70061)
Supplement: Supplementary file 1 [file NAP2-15-e70061-s001.docx]

**Supporting Information:**

**One-dimensional acoustic plasmons in metallic single-walled carbon nanotubes**

GyeongPyo Jeon^1^, Sihan Zhao^†^, In-Ho Lee^‡^ and SeokJae Yoo^*^

^*^Department of Physics, Program in Semiconductor and Device, Research and Education on Next-Generation Semiconductor Materials and Devices for Chiplet Technology, and Physics Research Institute, Inha University, Incheon, Republic of Korea

^†^School of Physics, Zhejiang Key Laboratory of Micro-Nano Quantum Chips and Quantum Control and State Key Laboratory of Silicon and Advanced Semiconductor Materials, Zhejiang University, Hangzhou 310058, China

^‡^Center for Quantum Technology, Korea Institute of Science and Technology, KIST, Seoul 02792, Republic of Korea

^1^Department of Physics, Inha University, Incheon, Republic of Korea

**1. Validity of the solid-cylinder model for metallic single-walled carbon nanotube**

In both the analytical and numerical modeling, the metallic single-walled nanotube is described as a solid cylindrical waveguide. Although a SWNT is physically a hollow cylinder formed by rolling a graphene sheet, modeling it as a solid cylinder is well justified for describing its plasmonic response for two reasons: (i) correlated electrons in SWNTs behave collectively rather than as quasi-free electrons residing on a hollow cylindrical surface, (ii) The large negative permittivity of the solid cylinder model screens the potential inside the cylinder yielding results equivalent to those of a hollow cylinder model as shown in Fig. S1. Historically, modeling SWNTs as solid cylinders in seminal work on Luttinger liquids [1]. More recent studies of Luttinger liquid plasmons in SWNTs [2, 3, 4] have also used the solid cylinder model, with predictions that match experimental results quite well. Notably, this solid versus hollow cylinder modeling question parallels debates in 2D van der Waals (vdW) materials, where disputes have arisen between 3D slab and 2D sheet models. The current consensus holds that these two models are equivalent when the vdW material is sufficiently thin compared to the wavelength of light. Analogously, since SWNT radius lies in the deep subwavelength regime (~1 nm compared to wavelengths of ~10 μm), both models can be considered valid.

Fig. S1: Numerical simulation of the electric-field distribution of the 1D acoustic plasmon supported by a SWNT. (a) Solid cylindrical model with radius *R* = 0.75 nm. (b) Shell model with the same outer radius *R* = 0.75 nm and shell thickness *d* = 0.34 nm.

**2. Even-order peak suppression in One-dimensional acoustic plasmons**

To clarify the behavior of even-odd peak oscillations in 1D acoustic plasmon systems, we calculated the plasmonic field profiles while varying the optical permittivity of the SWNT (Fig. S2). In Fig. S2, we present the plasmonic field amplitudes, Re(*E*_z_), calculated at the sharp tip end suspended 1 nm above the top surface of the SWNTs. For bare SWNTs freestanding in air (left panels in Fig. S2), the even-odd peak oscillation of 1D bare plasmons persists as the plasmon wavelength decreases from 111 nm (green curve) to 64 nm (orange curve), corresponding to *ε*_SWNT_ decreasing from -330+29.8i to -130+9.8i. In contrast, the even-odd peak oscillation is effectively suppressed in all 1D acoustic plasmons of the NToM (nanotube-on-a-mirror) configuration, as shown in the right panels of Fig. S2. Comparing plasmons with similar wavelengths, for example, 90 nm and 84 nm for the 1D bare (blue curve) and acoustic plasmons (green curve), respectively, the even-odd peak oscillation is markedly suppressed in 1D acoustic plasmons. Additionally, the field strengths in 1D acoustic plasmons are substantially stronger than those in 1D bare plasmons (Fig. S2). These observations support our interpretation that strong confinement leads to suppression of the even-odd peak oscillation.

**Fig. S2**: Plasmonic field Re(*E*_z_) of (a) 1D bare plasmons and (b) 1D acoustic plasmons with different SWNT permittivity. Re(*E*_z_) was calculated at the tip end of 1 nm-height.

**References**

[1]	C. Kane, L. Balents, and M. P. A. Fisher, “Coulomb Interactions and Mesoscopic Effects in Carbon Nanotubes,” Phys Rev Lett, vol. 79, no. 25, p. 5086, 1997, https://doi.org/10.1103/PhysRevLett.79.5086

[2]	Z. Shi et al., “Observation of a Luttinger-liquid plasmon in metallic single-walled carbon nanotubes,” Nature Photon 9, 515–519 (2015), https://doi.org/10.1038/nphoton.2015.123.

[3]	S. Yoo, S. Zhao, and F. Wang, “Infrared Light-Emitting Devices from Antenna-Coupled Luttinger Liquid Plasmons in Carbon Nanotubes,” Phys Rev Lett, vol. 127, no. 25, p. 257702, 2021, https://doi.org/10.1103/PhysRevLett.127.257702.

[4]	S. Wang et al., “Gate-tunable plasmons in mixed-dimensional van der Waals heterostructures,” Nature Communications 2021 12:1, vol. 12, no. 1, pp. 1–7, 2021, https://doi.org/10.1038/s41467-021-25269-0.

[5]	S. Wang et al., “Logarithm Diameter Scaling and Carrier Density Independence of One-Dimensional Luttinger Liquid Plasmon,” Nano Lett., vol. 19, no. 4, pp. 2360–2365, Apr. 2019, https://doi.org/10.1021/ACS.NANOLETT.8B05031.
